# Bilateral chylothorax following neck dissection: a case report

**DOI:** 10.1186/1756-0500-7-311

**Published:** 2014-05-22

**Authors:** Tina Runge, Yves Borbély, Daniel Candinas, Christian Seiler

**Affiliations:** 1 Department of Visceral Surgery and Medicine, Inselspital, University of Bern, Bern 3000, Switzerland

**Keywords:** Thyroidectomy, Neck dissection, Bilateral chylothorax

## Abstract

**Background:**

Chylothorax is an extremely rare but potentially life-threatening complication after radical neck dissection. We report the case of a bilateral chylothorax after total thyroidectomy and cervico-central and cervico-lateral lymphadenectomy for thyroid carcinoma.

**Case presentation:**

A 40-year-old European woman underwent total thyroidectomy and neck dissection for papillary thyroid carcinoma. Postoperatively she developed dyspnoea and pleural effusion. A chylothorax was found and the initial conservative therapy was not successful. She had to be operated on again and the thoracic duct was legated.

**Conclusion:**

The case presentation reports a very rare complication after total thyroidectomy and neck dissection, but it has to be kept in mind to prevent dangerous complications.

## Background

Chylous leaks after neck dissections is a known complication occurring in 1-2% of patients [1]. Chylothorax, however, is uncommon and bilateral chylothorax is extremely rare. So far, only 16 cases are reported in the literature. It is potentially life-threatening due to severe respiratory, metabolic and immunologic derangements.

We report the case of a bilateral chylothorax after total thyroidectomy with extended lymphadenectomy for thyroid carcinoma, successfully managed with ligation of the thoracic duct.

## Case presentation

A 40-year-old European woman presented with a 1-year history of a palpable anterior neck mass. Physical examination revealed a 1 × 1 cm indurated mass in the right thyroid lobe without palpable lymphadenopathy. Fine-needle aspiration confirmed a papillary thyroid carcinoma. She underwent a total thyroidectomy with cervico-central and cervico-lateral lymphadenectomy. Intraoperative histopathological examination revealed a bilateral papillary carcinoma pT1 pN1b. Laryngeal nerves and thoracic duct were identified and not harmed, and administration of cream via gastric tube showed no leakage. Two suction drains were left behind.

On the second postoperative day, the patient complained of dyspnoea and chest discomfort. In absence of neck swelling or suggestive fluid in her drains, a computed tomography of the chest for suspected pulmonary embolism was performed. Massive bilateral pleural effusions were detected; bilateral thoracic drainages were inserted and about 2 L of milky white fluid with a triglyceride level of 17 mmol/L were evacuated. Total parenteral nutrition with total enteral rest and octreotide were initiated. The output of both pleural drains was over 2 L a day. The patient was re-operated on post-operative day (pod) 4 due to the persisting thoracic discharge of over 2 L a day. Again, no chyle leak or injury to the thoracic duct was found. The thoracic duct was ligated. This resulted in a dramatic decrease in the drain output. The thoracic drains were removed on pod 9 and a regular diet was started again. The patient was discharged on pod 11.

The thoracic duct arises from the cisterna chyli at the level of the second lumbar vertebra. It enters at the base of the neck and curves to the left to cross the scalene anterior muscle and the thyrocervical trunk. From here it enters the internal jugular, left subclavian, left external jugular or right jugular vein with a high variability [2]. Injury to the duct usually occurs with thorough dissection of lymphatic tissue in this region. It is commonly recognized by milky effusion in the drain.

Chylothorax in absence of direct injury to the thoracic duct and therefore without chylous effusion however is rare. Bilateral chylothorax is even more uncommon. The pathophysiology is not completely understood; two pathologic mechanisms are discussed in the literature. First, direct leakage of the thoracic duct with subsequent drainage into the mediastinum leads to an inflammatory reaction that causes effusion to both thoracic cavities. Even though in this case no obvious injury to the thoracic duct was found intraoperatively, there has been reports of a few cases in which the suction drains in the thyroid cavity drained chyle. This has been thought to be an indirect sign of injury to the cervical lymphatic vessels.

The second pathologic mechanism is thought to be the inadvertent ligation of the thoracic duct which leads to a rise of the intraductal pressure intrathoracically. This rising pressure and in combination with the negative intrapleural pressure generated during inspiration is thought to be severe enough to result in the rupture of the thoracic duct. In our case study, we were not able to identify any injury to the large lymphatic vessels. In both initial and in the revision procedure, cream was administered and the thoracic duct was inspected. Furthermore, the thoracic duct was only ligated in the revision operation. Therefore, exact pathophysiology of chylothorax in our case study remains unknown.

Management of chylothorax is mostly conservative and is focused on decreasing the amount of chyle production and preventing concurrent nutritional, immunologic and cardiopulmonary complications.

The first measure is to drain the bilateral effusions by inserting chest tubes to relieve respiratory and circulatory distress. Diet comprising of middle chain fatty acids or even total parenteral nutrition without oral intake is recommended.

Administration of octreotide to reduce thoracic duct flow remains controversial [3].

Operative treatment is recommended for chyle leaks of more than 1 L per day persisting for more than 5 days or chylothorax remaining longer than 4 weeks, or in a case which results in severe metabolic complications.

However, some surgeons prefer early operative intervention to reduce hospital stay and morbidity secondary to long time nutritional restriction and drainage tubes [4]. Due to the paucity of cases, definitive treatment guidelines are still lacking.

Another aspect is the delay in treatment. This is particularly significant in oncologic patients where lymphadenectomies are being performed. In our patient, a computed chest tomography was performed due to a high clinical suspicion of a pulmonary embolic event. Due to iodide exposition, radioiodine therapy followed with a delay of 4 weeks.

## Conclusion

Bilateral chylothorax after neck dissection is a serious but rare complication. The underlying cause remains unclear. First-line therapy is conservative, and the current literature does not provide clear guidelines for necessity and timing of revisional surgery. Management will depend on the individual patient condition and complications from persisting chyle loss. A potential delay in commencing therapy in the case of carcinoma must also be kept in mind.

## Consent

Written informed consent was obtained from the patient for publication of this Case Report and any accompanying images. A copy of the written consent is available for review by the Editor-in-Chief of this journal.

## Abbreviation

pod: Post-operative day.

## Competing interests

The authors declare that they have no competing interests.

## Authors’ contributions

TR prepared the manuscript, and it was reviewed by YB, DC and CS. CS performed the thyrdoidectomy assisted by YB. All authors read and approved the final manuscript.

## Authors’ information

TR is a resident completing her basic surgical training in the Department of Visceral Surgery and Medicine, Inselspital, University of Bern. YB is a junior and CS a senior consultant in the Department of Visceral Surgery and Medicine, Inselspital, University of Bern. DC is the head of the Department of Visceral Surgery and Medicine, Inselspital, University of Bern.
